# First-line treatment outcomes in *HER2*-altered advanced NSCLC: a real-world multi-center study comparing trastuzumab deruxtecan with conventional therapies and analysis by *HER2*/*EGFR* co-alteration status

**DOI:** 10.1186/s12885-026-16688-7

**Published:** 2026-10-12

**Authors:** Chang Liu, Tao Li, Bo-yu Qin, Lu Yu, Chenchen Ji, Min Liu, An Wang, Yong-long Wang, Zhe-feng Liu, Tong Zhang, Yi Hu

**Affiliations:** 1 Senior Department of Oncology, Chinese PLA General Hospital, Beijing, 100029 China; 2 Medical School of Chinese PLA, Beijing, 100853 China; 3 School of Medicine, Nankai University, Tianjin, China; 4 Department of Stomatology, Chinese PLA General Hospital, Beijing, 100029 China; 5 Changchun Veterinary Research Institute, Key Laboratory of Jilin Province for Zoonosis Prevention and Control, Chinese Academy of Agricultural Sciences, Yujinxiang Street 573, Changchun, 130122 Jilin China; 6 Medical Laboratory Center, the First Hospital of Xinjiang Medical University, Urumqi, 830054 China; 7 Capital Medical University Electric Power Teaching Hospital, Beijing, 100071 China; 8 Department of Pharmacy, Medical Supplies Center of Chinese PLA General Hospital, Beijing, 100853 China

**Keywords:** Non-small cell lung cancer (NSCLC), *HER2*, *EGFR*, Co-alteration, Trastuzumab deruxtecan (T-DXd), First-line

## Abstract

**Background:**

First-line treatment options for *HER2*-altered advanced non-small cell lung cancer (NSCLC) remain limited, especially in cases with *HER2-EGFR* co-alterations. While new agents like trastuzumab deruxtecan (T-DXd) have emerged, its real-world evidence in first line remains limited.

**Methods:**

This study included patients with advanced NSCLC who had HER2 mutations or amplification and received first-line systemic therapy at six medical centers. Outcomes were evaluated following chemotherapy-based combinations, pan-HER tyrosine kinase inhibitors (TKIs), and T-DXd. Endpoints included progression-free survival (PFS), objective response rate (ORR), disease control rate (DCR), one-year and two-year survival rates, and toxicity.

**Results:**

A total of 69 patients were enrolled, comprising 45 with only *HER2* alterations and 24 with *HER2*-*EGFR* co-alterations. In patients with only *HER2*-altered NSCLC, those receiving first-line T-DXd had an mPFS of 15.7 months (95% CI, 12.5–18.9), an ORR of 85.7%, and a DCR of 100.0%. The mPFS for patients receiving chemotherapy-based regimens, pan-HER TKIs, and chemotherapy plus pan-HER TKIs were 7.6 (95% CI, 6.6–8.5) months, 4.4 (95% CI, 4.0-4.9) months, and 7.0 (95% CI, 6.1–9.1) months, respectively. The ORR for the three groups above was 33.3%, 0, and 0, respectively. In subgroup analysis, patients with only *HER2* alterations who did not harbor *TP53* mutations had a longer PFS than those with *TP53* co-mutations (8.7 m vs. 7.2 m; HR: 0.35; 95% CI: 0.11–0.99; *P* = 0.048). In patients with *EGFR-HER2* co-alterations, first-line EGFR-TKI monotherapy yielded an mPFS of 9.7 (95% CI, 8.6–10.8) months, an ORR of 40.0%, and a DCR of 93.3%. Regarding safety, we did not observe Grade ≥ 3 TRAEs in first-line T-DXd treatment.

**Conclusions:**

First-line T-DXd showed promising activity signals in only *HER2*-altered NSCLC. In *EGFR-HER2* co-altered patients, the EGFR-TKI monotherapy showed limited efficacy. The findings underscored the urgent need for prospective randomised trials.

**Supplementary Information:**

The online version contains supplementary material available at https://doi.org/10.1186/s12885-026-16688-7.

## Introduction

*HER2* alterations drive tumorigenesis in various cancers [1], primarily as mutations, amplification, or protein overexpression [2]. In non-small lung cancer (NSCLC), *HER2* is a rare but important driver gene [3]. NSCLC constitutes 85% of lung cancer cases [4], and advanced-stage 5-year survival remains below 10% [5]. In Chinese patients, *HER2* mutations and amplifications occur in 2–4% and 1.7% of NSCLC, respectively [6]. For advanced *HER2*-mutant NSCLC, the National Comprehensive Cancer Network (NCCN) guidelines recommend first-line platinum-based chemotherapy ± immunotherapy before 2026 [7]. First-line chemotherapy offers limited benefit (mPFS: 4.4 months), slightly improved with added immunotherapy (mPFS: 8.2 months) [8]. Prior therapies, including monoclonal antibodies, pan-*HER* TKIs, and T-DM1, showed limited efficacy [9–13]. In February 2026, the Food and Drug Administration (FDA) approved Zongertinib for first-line treatment of such patients [14]. Post-progression, both NCCN and the Chinese Society of Clinical Oncology (CSCO) guidelines prioritised the ADC trastuzumab deruxtecan (T-DXd). And in 2025, the FDA successively approved Zongertinib [15] and Sevabertinib [16] for the treatment of patients with advanced NSCLC harboring *HER2* mutations who had previously received systemic therapy. However, for patients with *HER2* non-TKD mutations, amplifications, or brain metastases, T-DXd remained a viable option [17].

ADCs represent an emerging class of anticancer agents [18]. Currently, T-DXd is the first ADC approved by the FDA for the treatment of HER2-mutant advanced NSCLC [19]. Its superior efficacy and manageable safety profile in DESTINY-Lung05 [20] have established T-DXd as the standard second-line therapy for this patient group. However, these data were derived from later-line settings. The efficacy and safety of T-DXd, and indeed all ADCs, in the first-line treatment of *HER2*-altered advanced NSCLC remained unexplored. The ongoing DESTINY-Lung04 trial aims to compare T-DXd with chemoimmunotherapy in the first-line setting for advanced *HER2*-mutant NSCLC, though no results have been reported to date.

*HER2* alterations are a key mechanism of resistance to EGFR-TKIs [21]. The emergence of such “secondary” co-alterations under therapeutic pressure underscores the important role of the *HER2* pathway in escaping *EGFR* inhibition. For tumors harboring a “primary” HER2-EGFR co-alteration before first-line treatment, a remaining unanswered question is whether this inherent dual-pathway activation leads to primary or rapid adaptive resistance to EGFR-TKIs. Currently, due to the relative rarity of these co-alterations, this specific molecular subgroup has been documented only in case reports [22], with no prospective or basic research. Based on the FLAURA trial [22], the third-generation EGFR-TKI osimertinib has become the first-line standard of treatment for *EGFR*-mutant advanced NSCLC. The FLAURA2 trial [23] investigated an intensification strategy of osimertinib plus chemotherapy, confirming a significant extension in mPFS for the overall population. However, both studies focused on the overall *EGFR*-mutant population without specifying whether HER2-EGFR co-alterations were present.

This real-world, multicenter study compared treatment outcomes in NSCLC with *HER2* alterations, with a specific focus on the *HER2*-*EGFR* co-alteration subtype, to inform future precision therapy research.

## Methods

### Patient population and study design

**Fig. 1:**
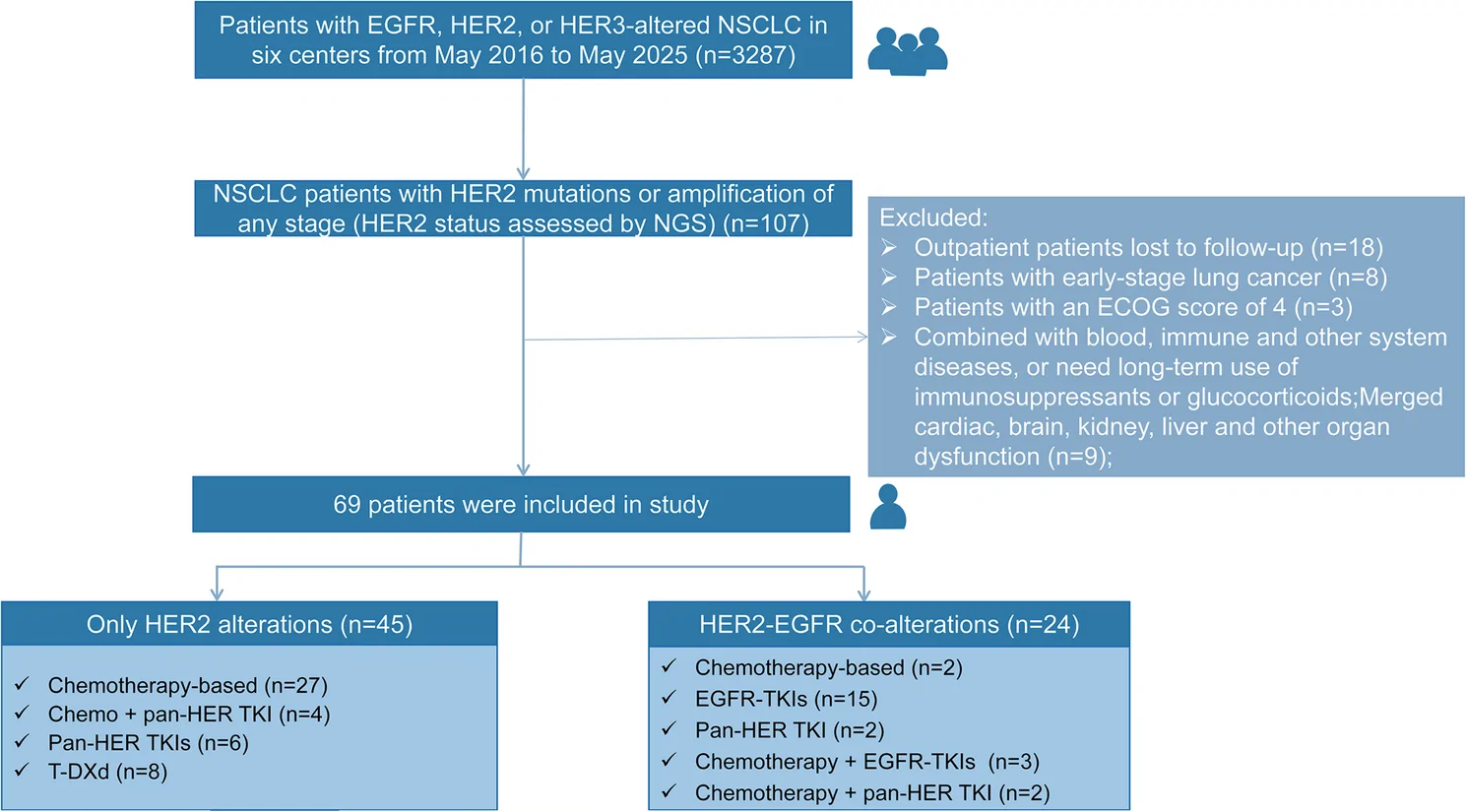
Diagram of this study

This multicenter retrospective study included patients with advanced NSCLC and HER2 alterations who received first-line systemic anti-tumour therapy between January 2016 and December 2025 at the First, Fourth, Fifth, Sixth, Seventh, and Eighth Medical Centres of PLA General Hospital. Inclusion criteria required pathologically confirmed NSCLC, age ≥ 18 years, and *HER2* mutation or amplification (defined as *HER2* copy number ≥ 6 [24]) confirmed by next-generation sequencing (NGS) of tumor tissue or liquid biopsy samples. Currently, there are no FISH detection criteria for *HER2* amplification in NSCLC, and FISH testing has not been included in China as a recommended diagnostic test at diagnosis. Therefore, in our final cohort, FISH was not used, and all amplifications were determined by NGS. Specific inclusion and exclusion criteria are detailed in Fig. 1. *HER2*-*EGFR* co-alteration was defined as the simultaneous presence of activated *EGFR* mutations and *HER2* alterations (mutations or amplifications) in the same test report detected by NGS before first-line therapy. Post-treatment acquired alterations were excluded from the cohort. When both alterations were detected simultaneously in the same tissue specimen, it was classified as tissue confirmation. When the co-alterations were detected only by plasma ctDNA, it was classified as plasma-defined. Since it could not be determined whether the two alterations originated from the same tumor clone, such cases were treated separately in sensitivity analyses. In cases where tissue and plasma results were inconsistent, the tissue result shall prevail.

A total of 69 eligible patients were enrolled. Among them, 45 patients (65.2%) had only *HER2* alterations, and 24 patients (34.8%) had *HER2*-*EGFR* co-alterations. Clinicopathological characteristics and treatment outcomes were collected, including age, gender, smoking history, ECOG performance status, TNM stage, histology, metastatic sites, *HER2* alteration type, comprehensive genomic profiling results, treatment regimens, outcomes, and adverse events.

The preliminary analysis of this study included both stage III and stage IV patients. It should be noted that for unresectable stage IIIB/IIIC NSCLC, the standard treatment regimen consists of concurrent chemoradiotherapy followed by sequential consolidation therapy with durvalumab. In our cohort, all 13 Stage III patients were deemed unresectable and ineligible for curative-intent chemoradiotherapy due to one or more of the following reasons: poor performance status and intolerant to radiotherapy (ECOG 2 or 3, *n* = 5), extensive mediastinal lymph node involvement precluding safe radiotherapy (*n* = 4), and patient refusal of radiotherapy (*n* = 4). Consequently, these patients received systemic therapy as a palliative or alternative approach, which, while not standard, reflected real-world clinical practice for this challenging subgroup. To evaluate the impact of including patients with stage IIIB/IIIC on the results, we conducted a sensitivity analysis (excluding all stage IIIB/IIIC patients). We reported the survival outcomes stratified by stage in Supplement Table S1.

During the study period, T-DXd was approved for the treatment of previously treated advanced *HER2*-mutant NSCLC. Specifically, the National Medical Products Administration (NMPA) of China approved T-DXd in this indication in October 2024. While a case report has indicated its efficacy in the first-line setting [25], larger-scale validation is needed. Treatment allocation was neither randomised nor protocol‑driven. During the study period, treatment decisions were made jointly by patients and physicians after a comprehensive discussion of tumor burden, ECOG performance status, patient preference, financial considerations, drug accessibility, and available clinical evidence. Documented justifications, as extracted from medical records, included: (1) refusal of platinum-based chemotherapy due to toxicity concerns; (2) poor performance status considered borderline for chemotherapy tolerance; and (3) strong patient preference based on evidence from the DESTINY-Lung05 trial. These selection factors introduced a bias that must be considered when comparing outcomes to other treatment groups. Given the retrospective nature of the study and the use of de-identified data, the Institutional Review Board (IRB) waived the requirement for written informed consent (ethics approval number: S2024-794).

### Efficacy assessment of treatment

This study assessed treatment efficacy according to Response Evaluation Criteria in Solid Tumors (RECIST) version 1.1. Tumor response was assessed retrospectively based on original radiology reports from routine clinical practice. All reports were generated by board-certified radiologists at each participating center. No independent central review was performed. Outcome measures included progression-free survival (PFS), overall survival (OS), objective response rate (ORR), disease control rate (DCR), and one-year and two-year survival rates. PFS was defined as the time from treatment initiation to disease progression or death from any cause; OS as the time from treatment initiation to death from any cause; ORR as the proportion of patients achieving a complete response (CR) or partial response (PR); DCR as the proportion achieving CR, PR, or stable disease (SD). The one-year and two-year survival rates were defined as the proportion of patients alive beyond one and two years after treatment, respectively. All patients were followed closely until December 1, 2025, or death, ensuring a comprehensive follow-up period for analysis.

### Adverse events assessment

All adverse event (AE) data in this study were retrospectively collected from electronic medical records, nursing notes, laboratory tests, and imaging reports. The AE evaluation period ranged from the initiation of first-line therapy until 30 days after the last dose or death, whichever occurred first. AE grading was performed according to CTCAE 5.0 based on recorded clinical and laboratory findings. As the study was retrospective in design, we did not conduct prospective, protocol-mandated AE monitoring. Particular attention was paid to treatment-related adverse events (TRAEs), all of which were documented and graded in detail.

### Statistical analysis

All statistical analyses were performed using SPSS version 29.0 and R Studio version 4.0.5. Categorical data were presented as numbers and percentages, while continuous variables were expressed as the median with range or the mean with standard deviation. Survival analysis was conducted using the Kaplan-Meier method and the Log-rank test (two-sided). Univariate and multivariate analyses for hazard ratios (HRs) with 95% confidence intervals (CIs) were performed using the Cox proportional hazards regression model. Given the retrospective design and the small sample size in each treatment group, all comparisons were exploratory. P values and HRs were reported only to describe the direction and uncertainty of association and were not used to infer superiority.

## Results

### Patient demographics and characteristics

This study included 69 patients with advanced NSCLC harboring *HER2* mutations or amplifications **(**Fig. 1**)**. Among them, 45 patients had only *HER2* alterations, and 24 patients had *HER2*-*EGFR* co-alterations. Detailed demographic data of the patients were presented in Table 1. The median age of the patients was 59 years (range, 35–80). The cohort comprised 31 male (44.9%) and 38 female patients (55.1%), and 68.1% had no history of smoking. Regarding ECOG performance status, 87% of patients had a score of 0 or 1, indicating good physical condition before first-line therapy for advanced disease. Pathologically, the vast majority of patients had adenocarcinoma (98.6%). At diagnosis, 13 patients (18.8%) were classified as stage IIIB or IIIC, while the remaining 56 patients had stage IV disease. Tumor differentiation was evaluable in 39 patients (56.5%). Among them, 25 patients (36.2%) had poorly differentiated or moderately to poorly differentiated tumors, and only 2 patients (2.9%) were considered well-differentiated.

**Table 1:** Baseline characteristics of *HER2*-altered NSCLC patients

Characteristics	Only *HER2* alterations (*n* = 45)	*HER2-EGFR* co-alterations (*n* = 24)	Total(*n* = 69)	*P* value
Sex				0.260
Male	18(40.0)	13(54.2)	31(44.9)	
Female	27(60.0)	11(45.8)	38(55.1)	
Year				0.976
Y ≤ 50	10(22.2)	5(20.8)	15(21.7)	
50 < Y ≤ 65	25(55.6)	14(58.3)	39(56.5)	
Y > 65	10(22.2)	5(20.8)	15(21.7)	
Smoking history				0.203
Never	33(73.3)	14(58.3)	47(68.1)	
Current/former	12(26.7)	10(41.7)	22(31.9)	
ECOG				0.515
0	9(20.0)	5(20.8)	14(20.3)	
1	28(62.2)	18(75.0)	46(66.7)	
2	7(15.6)	1(4.2)	8(11.6)	
3	1(2.2)	0(0)	1(1.4)	
Pathology				1.000
Adenocarcinoma	44(97.8)	24(100.0)	68(98.6)	
Large cell carcinoma	1(2.2)	0(0)	1(1.4)	
Differentiation				0.564
NA	22(48.9)	8(33.3)	30(43.5)	
poorly differentiation	9(20.0)	6(25.0)	15(21.7)	
Moderately to poorly differentiated	7(15.6)	3(12.5)	10(14.5)	
Moderately differentiation	6(13.3)	6(25.0)	12(17.4)	
Moderately to well differentiated	1(2.2)	1(4.2)	2(2.9)	
Metastases				
Lung	38(84.4)	20(83.3)	58(84.1)	1.000
Bone	17(37.8)	12(50.0)	29(42.0)	0.327
Liver	10(22.2)	2(8.3)	12(17.4)	0.194
Brain	8(17.8)	2(8.3)	10(14.5)	0.475
Pleural effusion	11(24.4)	6(25.0)	17(24.6)	0.959
UICC stage				0.853
ⅢB/ⅢC	8(17.8)	5(20.8)	13(18.8)	
ⅣA	17(37.8)	10(41.7)	27(39.1)	
ⅣB	20(44.4)	9(37.5)	29(42.0)	
*HER2* mutations				< 0.001
NA	2(4.4)	6(25.0)	8(11.6)	
EX20ins mutations	29(64.4)	1(4.2)	30(43.5)	
Missense mutations	6(13.3)	9(37.5)	15(21.7)	
Fusion mutations	1(2.2)	1(4.2)	2(2.9)	
Frameshift mutations	2(4.4)	1(4.2)	3(4.3)	
Amplification	10(22.2)	10(41.7)	20(29.0)	0.065
PD-L1 expression				0.720
NA	18(40.0)	12(50.0)	30(43.5)	
Negative	13(28.9)	4(16.7)	17(24.6)	
1%≤ <50%	10(22.2)	6(25.0)	16(23.2)	
≥ 50%	4(8.9)	2(8.3)	6(8.7)	
TMB				0.735
NA	23(51.1)	15(62.5)	38(55.1)	
< 10	14(31.1)	6(25.0)	20(29.0)	
≥ 10	8(17.8)	3(12.5)	11(15.9)	
*HER2* alteration partner				
* TP53*	17(37.8)	15(62.5)	32(46.4)	0.050
Cell cycle-related gene mutations	8(17.8)	2(8.3)	10(14.5)	0.475
Treatment				<0.001
Chemo	27(60.0)	2(8.3)	29(42.0)	
Chemo+TKIs	4(8.9)	5(20.8)	9(13.0)	
TKIs	6(13.3)	17(70.8)	23(33.3)	
ADC	8(17.8)	0(0)	8(11.6)	

The most common sites of metastasis at initial diagnosis were the lung, bone, and malignant pleural effusion in both groups. Central nervous system metastases were present in 17.8% of patients with only *HER2* alteration and 8.3% of patients with *HER2*-*EGFR* co-alteration.

### Molecular profile

*HER2* alterations were assessed in 52 patients (75.4%) using tumor tissue and in 17 patients (24.6%) using plasma. NGS identified 20 distinct *HER2* mutations, comprising 3 insertion types, 14 missense mutations (*n* = 15, 21.7%), 2 fusion mutations, and 1 splice-site mutation.

**Fig. 2:**
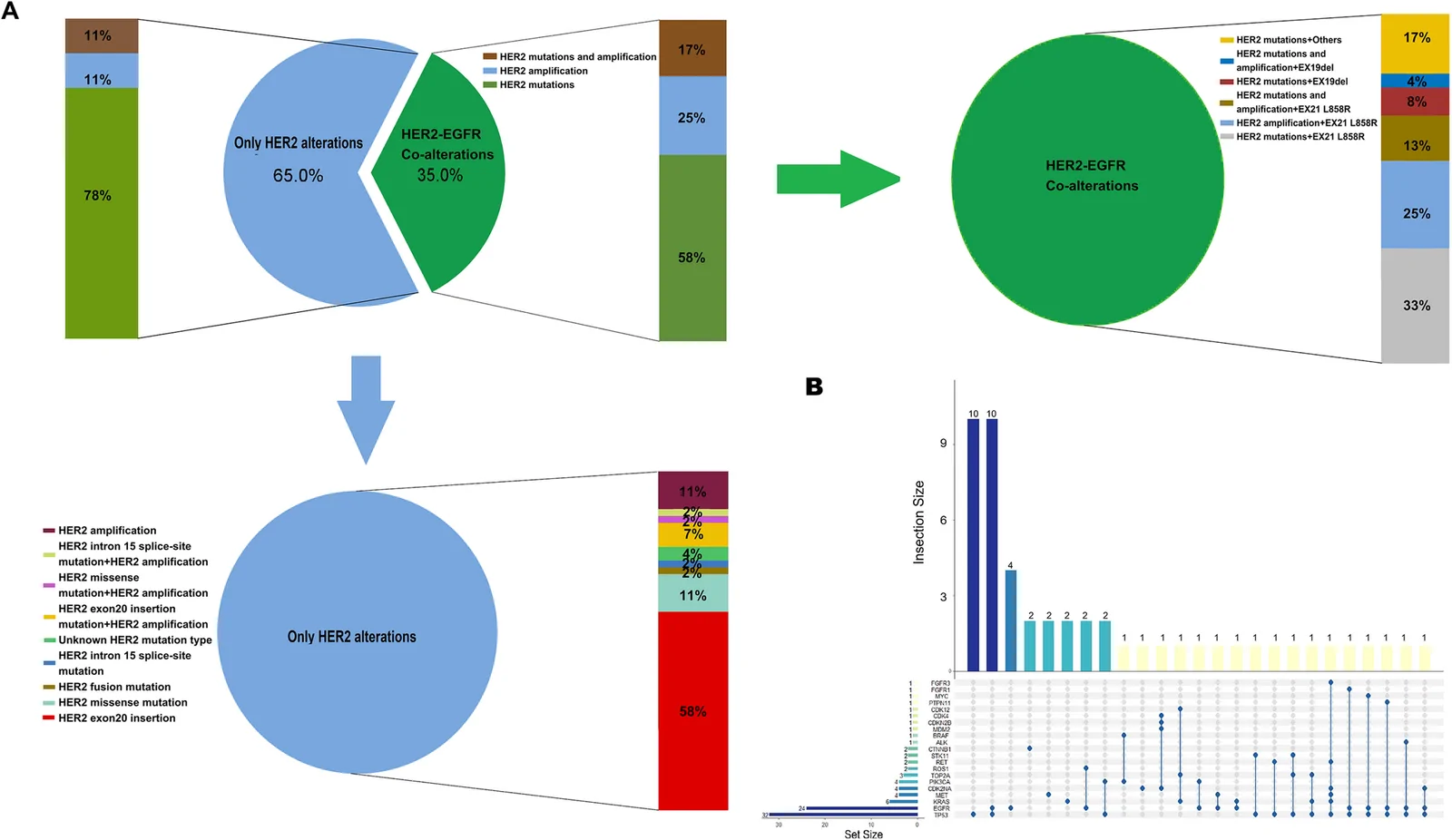
Molecular profile of *HER2*-altered NSCLC. **A** Concomitant altered genes together in NSCLC patients with *HER2* alterations detected using NGS among patients at primary diagnosis, as per the Venn plot. **B** Distribution of different gene alteration types in patients with baseline *HER2* alterations

In patients with only *HER2* alteration, the most common *HER2* mutation subtype was exon 20 insertion mutations (*n* = 29, 64.4%) **(**Fig. 2A**)**. Among these, the specific insertion site was undetermined for 7 patients. The A775_G776insYVMA mutation was most frequent (*n* = 17, 37.8%), followed by G776delinsVC (*n* = 4, 8.9%), and one case of P780_Y781insGSP. In terms of amplification, in addition to pure HER2 amplification (*n* = 5, 11.1%), HER2 co-occurring alterations were observed in 5 patients: 3 with exon 20 insertions plus amplification, 1 with missense mutations plus amplification, and 1 case each of a splice-site mutation with amplification. Within the cohort, *TP53* was the most common co-mutation (*n* = 17, 37.8%), and mutations in cell cycle pathway proteins, including *TOP2A*,* CDKN2A*,* CDKN2B*,* CDK4*, and *CDK12*, were present in 8 patients (17.8%).

In patients with *HER2*-*EGFR* co-alteration, the *HER2* mutation subtype was undetermined for 6 patients. Besides, the most common *HER2* mutation subtype was missense mutations (*n* = 9, 37.5%), of which 3 patients exhibited *HER2* amplification **(**Fig. 2B**)**. One patient each with an exon 20 insertion mutation and a fusion mutation. In terms of amplification, in addition to pure HER2 amplification (*n* = 6, 25.0%), HER2 co-occurring alterations were observed in 4 patients: 3 with missense mutations plus amplification and 1 with a fusion mutation plus amplification. Within the cohort, *TP53* was the most common co-mutation (*n* = 15, 62.5%). Among *EGFR* mutations, 18 patients harbored an exon 21 L858R mutation, 3 had an exon 19 deletion, 2 exhibited an exon 20 insertion mutation, and 1 showed an exon 24 M952K mutation. Mutations in cell-cycle pathway proteins were detected in 2 patients (8.3%).

### Regimens and efficacy

**Fig. 3:**
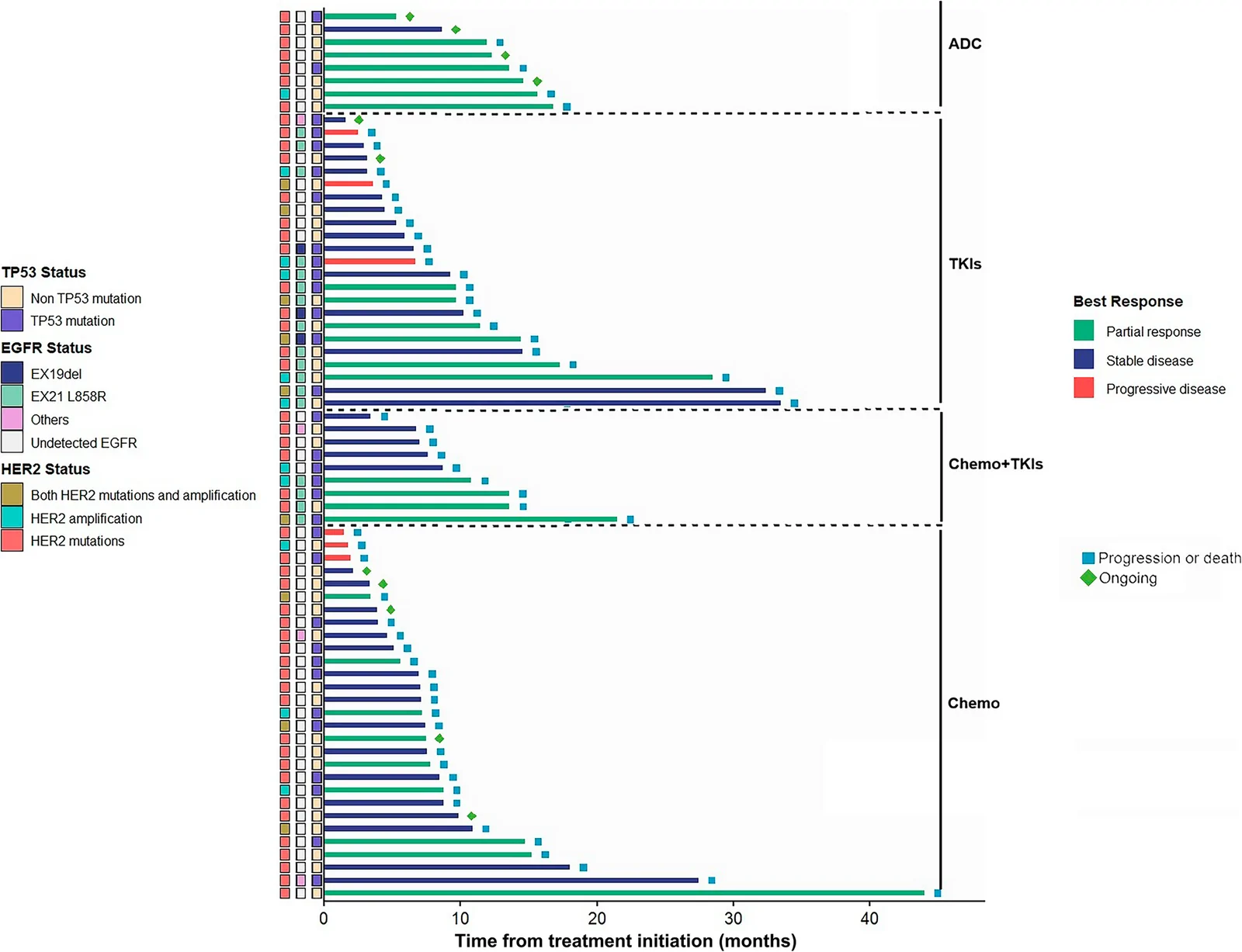
Treatment details. A Swimmer’s plot of duration of first-line therapy after detection of *HER2* alterations

Among the 69 patients available for first-line treatment efficacy analysis, 58 patients had PFS events **(**Fig. 3**)**, and only 27 patients (39.1%) had OS events. The median follow-up duration was 28.9 (95% CI: 19.6–38.2) months. Therefore, we refrained from making any inferences regarding OS benefit and reported one-year and two-year survival rates solely for descriptive purposes.

#### Patients with advanced NSCLC harboring only *HER2* alterations

**Fig. 4:**
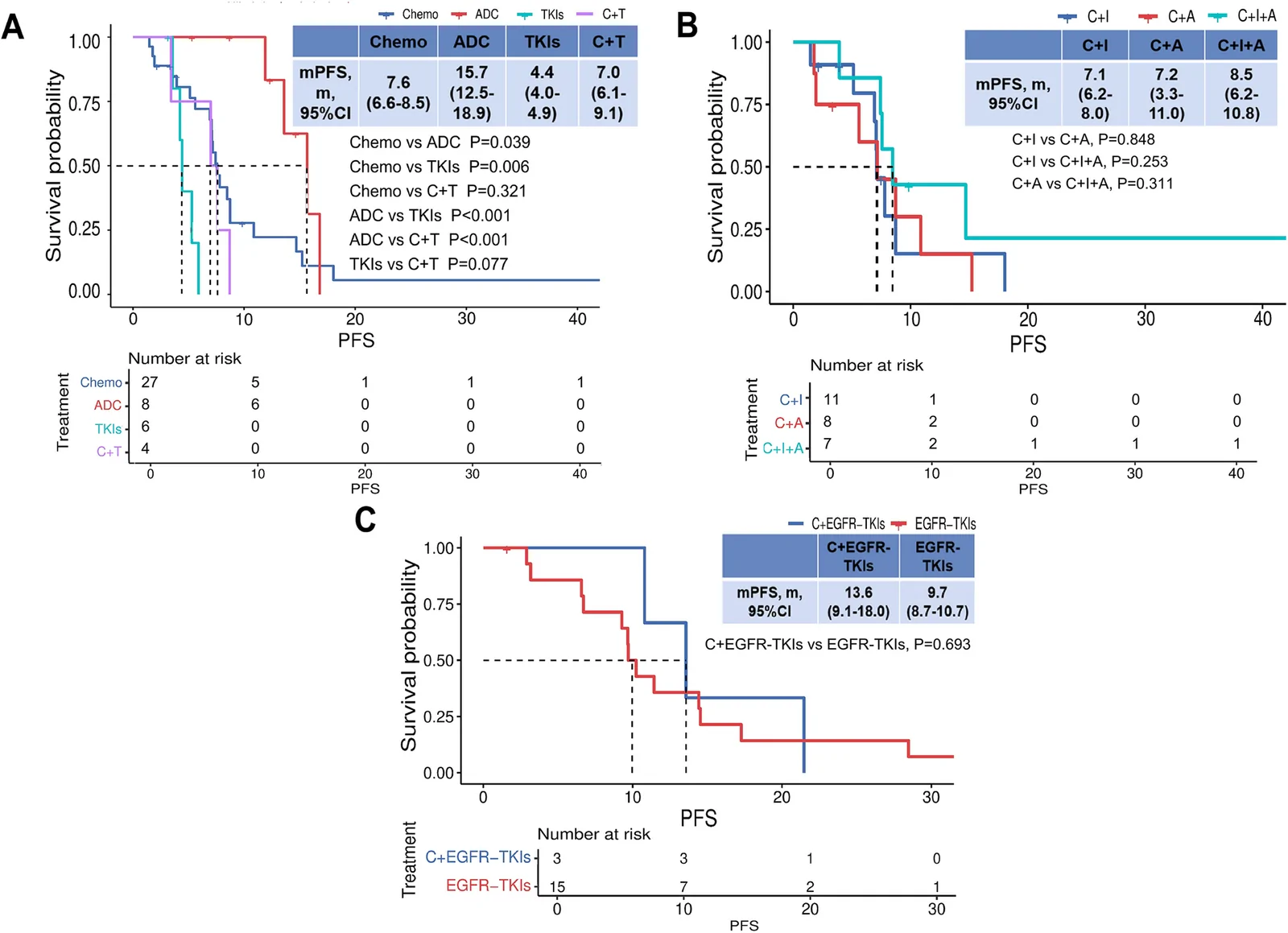
Kaplan-Meier estimates of PFS in patients with NSCLC harboring only *HER2* alterations or *HER2*-*EGFR* co-mutations treated in the first-line setting. **A** PFS in patients harboring only *HER2* alterations receiving chemotherapy, ADCs, TKIs, and chemotherapy combined with TKIs (C + T). **B** PFS in patients harboring only *HER2* alterations receiving chemotherapy combined with ICIs (C + I), chemotherapy combined with anti-angiogenic agents (C + A), and chemotherapy combined with both ICIs and anti-angiogenic agents (C + I+A). **C** PFS in patients harboring *HER2*-*EGFR* co-alterations treated with EGFR-TKIs and chemotherapy combined with EGFR-TKIs (C + EGFR-TKIs)

Among the 45 patients with only *HER2* alterations, 5 had pure *HER2* amplification (Supplement Table S2). First-line treatments were: chemotherapy-based (*n* = 27); chemotherapy plus pan-HER TKI (C + T, *n* = 4; all received pyrotinib); pan-HER TKI monotherapy (*n* = 6; dacomitinib, *n* = 2; afatinib, *n* = 4); and ADC therapy (*n* = 8; all received T-DXd). The chemotherapy-based group comprised chemotherapy alone (*n* = 1), chemoimmunotherapy (C + I, *n* = 11), chemotherapy plus antiangiogenic therapy (C + A, *n* = 8), and chemoimmunotherapy plus antiangiogenic therapy (C + I+A, *n* = 7). The mPFS for the chemotherapy-based group was 7.6 (95%CI, 6.6–8.5) months, with an ORR of 33.3% **(**Fig. 4A**)**. Patients in the C + I+A group had a longer mPFS (8.5 months, 95% CI, 6.2–10.8) than those in the C + I group (7.1 months, 95% CI, 6.2-8.0) and the C + A group (7.2 months, 95% CI, 3.3–11.0) **(**Table 2**)**, but differences were not statistically significant **(**Fig. 4B**)**.

**Table 2:** Clinical activity of different therapies in *HER2*-altered advanced NSCLC patients

	Only HER2 alterations						HER2-EGFR co-alterations	
	C + I (*n* = 11)	C + A (*n* = 8)	C + I+A (*n* = 7)	C + T (*n* = 4)	Pan-HER TKIs (*n* = 6)	T-DXd (*n* = 8)	EGFR-TKIs (*n* = 15)	Chemotherapy + EGFR-TKIs (*n* = 3)
Best Response, n (%)								
CR	0	0	0	0	0	0	0	0
PR	2	4	2	0	0	7	6	3
SD	8	2	5	4	5	1	8	0
PD	1	2	0	0	1	0	1	0
ORR, %	2(18.2)	4(50.0)	2(28.6)	0(0)	0(0)	7(87.5)	6 (40.0)	3(100)
DCR, %	10(90.9)	6(75.0)	7(100)	4(100)	5(83.3)	8(100)	14 (93.3)	3(100)
PFS								
Events, n (%)	8(72.7)	7(87.5)	6(85.7)	4(100.0)	5(83.3)	4(50.0)	14 (93.3)	3(100)
Median, months (95% CI)	7.1 (6.2-8.0)	7.2 (3.3–11.0)	8.5 (6.2–10.8)	7.0 (6.1–9.1)	4.4 (4.0-4.9)	15.7 (12.5–18.9)	9.7 (8.6–10.8)	13.6 (9.1–18.0)
One-year survival rate, %	11 (100)	7 (87.5)	7 (100)	4 (100)	6 (100)	8 (100)	15 (100)	3(100)
Two-year survival rate, %	11 (100)	6 (75.0)	7 (100)	3 (75.0)	2(33.3)	NE	13 (86.7)	3(100)

In the ADC group (all received T-DXd), the ORR and DCR were 87.5% and 100%, respectively, with an mPFS of 15.7 (95% CI, 12.5–18.9) months. Among the 8 patients who received first-line T-DXd, 7 achieved a PR. The median time to first response (defined as the time from randomisation to the first occurrence of a CR or PR per RECIST v1.1) was 1.7 (95%CI, 1.4-2.0) months. At the data cutoff, the two-year OS rate was not mature, as only two deaths had occurred; the one-year OS rate was 100%. The mPFS was 7.0 (95% CI, 6.1–9.1) months for the C + T group (all patients received pyrotinib plus chemotherapy). The TKI monotherapy group (*n* = 6) received pan-HER TKIs, including dacomitinib (*n* = 2) and afatinib (*n* = 4), with an mPFS of 4.4 (95% CI, 4.0-4.9) months.

In patients with only *HER2* alterations, the ADC group showed a numerically longer mPFS than the chemotherapy group (median, 15.7 vs. 7.6 months, exploratory *P* = 0.039), the C + T group (median, 15.7 vs. 7.0 months, exploratory *P* < 0.001), and the pan-HER TKI group (median, 15.7 vs. 4.4 months, exploratory *P* < 0.001) **(**Fig. 4A**)**.

Importantly, the pan-HER TKIs used in our cohort (afatinib, dacomitinib) are distinct from the newer selective *HER2* TKIs. Thus, our observed poor outcomes with pan-HER TKI monotherapy should not be extrapolated to agents such as zongertinib or sevabertinib.

#### Patients with advanced NSCLC harboring *HER2*-*EGFR* co-alterations

This study included 24 patients with *HER2*-*EGFR* co-alterations, of whom 6 had pure *HER2* amplification (Supplement Table S3). First-line treatment comprised chemotherapy alone (*n* = 2), EGFR-TKI monotherapy (*n* = 15), chemotherapy plus EGFR-TKI (*n* = 3), pan-HER TKI monotherapy (dacomitinib, *n* = 2), and chemotherapy plus Pan-HER TKI (afatinib, *n* = 2). For EGFR-TKI monotherapy, mPFS was 9.7 (95% CI, 8.6–10.8) months, with ORR and DCR of 40.0% and 93.3%, respectively. Subgroups included first-generation (*n* = 7) and third-generation (*n* = 8) EGFR-TKIs. mPFS was similar between first- and third-generation EGFR-TKIs (9.7 vs. 10.2 months, exploratory *P* = 0.930).

The mPFS for the EGFR-TKI + chemotherapy group (*n* = 3) was 13.6 (95%CI, 9.1-18.0) months, while an estimate was not feasible for the pan-HER TKI + chemotherapy group (*n*= 2) due to the small sample size. Among the 15 patients receiving EGFR-TKI monotherapy and the 3 patients receiving EGFR-TKI + chemotherapy, the mPFS was 9.7 months and 13.6 (95%CI, 9.1–18.0) months, respectively (exploratory *P* = 0.693) **(**Fig. 4C**)**. Given that only 3 cases were included in the combined chemotherapy group, the statistical power was severely insufficient. Thus, these results cannot rule out potential benefits or risks of the combination regimen, necessitating further studies with larger sample sizes.

### Subgroup analysis

#### *HER2* alteration subtype

Among the 45 patients with only *HER2* alterations, there were 35 with pure *HER2* mutations (including 26 exon 20 insertions, 5 missense mutations, 1 fusion, 1 splice-site mutation, and 2 with unknown mutation sites), 5 with pure *HER2* amplification, and 5 with concurrent mutation and amplification. For the 35 patients with pure *HER2* mutations, the overall mPFS was 7.8 (95% CI, 6.1–9.5) months, with an ORR of 34.3% and DCR of 94.3%. Among these, 21 received chemotherapy-based regimens, 3 received chemotherapy plus pyrotinib, 4 received pan-HER TKI monotherapy, and 7 received T-DXd. The ORR were 28.6%, 0%, 0%, and 85.7%, respectively. The DCRs were 90.5%, 0%, 0%, and 100.0%. The mPFS was 7.8 (95% CI, 6.5–9.1) months, 7.0 (95% CI, 1.2–12.8) months, 5.3 (95% CI, 3.5–7.1) months, and 16.8 (95% CI, NE-NE) months, respectively. Compared with chemotherapy-based therapy (16.8 vs. 7.8 months, exploratory *P* = 0.124), chemotherapy plus pyrotinib (16.8 vs. 7.0 months, exploratory *P* = 0.001), and pan-HER TKI monotherapy (16.8 vs. 5.3 months, exploratory *P* = 0.001), T-DXd showed a numerically longer PFS (Supplement Figure S1A).

Among the 26 patients with *HER2* exon 20 insertions, the overall mPFS was 7.6 (95% CI, 6.6–8.6) months. Of these, 14 received chemotherapy-based regimens, 3 received chemotherapy plus pyrotinib, 3 received pan-HER TKI monotherapy, and 6 received T-DXd. The mPFS were 7.6 (95% CI, 6.9–8.3) months, 7.0 (95% CI, 1.2–12.8) months, 5.3 (95% CI, NE-NE) months, and 16.8 (95% CI, NE-NE) months, respectively. Again, T-DXd showed a numerically longer PFS compared to chemotherapy (16.8 vs. 7.1 months, exploratory *P* = 0.050), chemotherapy plus pyrotinib (16.8 vs. 7.0 months, exploratory *P* = 0.003), and pan-HER TKI monotherapy (16.8 vs. 4.4 months, exploratory *P* = 0.006) (Supplement Figure S1B). The 5 patients with *HER2* missense mutations all received chemotherapy-based regimens, with the mPFS of 8.7 (95% CI, NE-18.6) months. Due to very small sample sizes, the pure *HER2* amplification and mutation-plus-amplification subgroups were not analysed separately.

In the *HER2*-*EGFR* co-alteration cohort, numerically similar mPFS (median, 10.8 vs. 10.2 months; exploratory *P* = 0.099) were observed by amplification status (Supplement Figure S1C).

#### PD-L1 expression analysis

In the only *HER2*‑altered group, after excluding the one patient who received chemotherapy alone, 26 of 44 patients (59.1%) had documented PD‑L1 results. The distribution of PD‑L1 expression across treatment groups was generally balanced (*P* = 0.332, Fisher’s exact test), as shown in Supplementary Table S4. We further explored the association between PD‑L1 expression and prognosis. No significant difference in PFS was observed between PD‑L1‑negative patients (TPS < 1%) and those with PD‑L1‑positive expression (TPS ≥ 1%) (8.7 months vs. 5.9 months, exploratory *P* = 0.234) (Supplement Figure S1D). However, these exploratory analyses were limited by small sample sizes and incomplete data.

#### *TP53* mutation

In the group with only *HER2* alterations, 28 patients did not harbor TP53 mutations, and 17 did. Their mPFS was 8.7 (95% CI, 4.2–13.2) months and 7.2 (95% CI, 4.9–9.5) months, respectively (K-M exploratory *P* = 0.009) (Supplement Figure S1E). The HR between these groups was 0.39 (95% CI, 0.19–0.81) (*P* = 0.012). After adjusting for age, gender, ECOG performance status, tumor stage, metastatic sites, treatment modality, and type of *HER2* alteration, a multivariate Cox regression analysis yielded an HR of 0.35 (95% CI, 0.11–0.99) (*P* = 0.048).

In the *HER2*-*EGFR* co-modified group, 9 patients did not harbor TP53 mutations, and 15 did. Their mPFS was 13.6 (95% CI, 7.3–19.9) months and 9.7 (95% CI, 7.9–11.4) months, respectively (K-M exploratory *P* = 0.260) (Supplement Figure S1F). The HR was 0.61 (95% CI, 0.25–1.46) (*P* = 0.265). After multivariate regression analysis, the HR decreased to 0.14 (95% CI, 0.03–0.65) (*P* = 0.011).

### Sensitivity analysis

#### Patients with stage IVA and stage IVB

To assess potential confounding effects of including NSCLC patients with unresectable stage IIIB/IIIC disease, we conducted a sensitivity analysis. We reanalysed the outcome after excluding all 13 stage III patients.

In the only *HER2*-altered subgroup (*n* = 37), the ORR for patients receiving chemotherapy-based therapy (*n* = 20), chemotherapy plus pan-HER TKIs (*n* = 4, all received pyrotinib), pan-HER TKIs monotherapy (*n* = 5, 1 received dacomitinib and 4 received afatinib), and T-DXd therapy (*n* = 8) were 35.0%, 0%, 87.5%, respectively. The DCR rates were 85.0%, 100%, 80.0%, and 100%. The mPFS were 7.6 (95% CI: 6.6–8.5) months, 7.0 (95% CI: 2.9–11.1) months, 4.2 (95% CI: 3.4–5.1) months, and 15.7 (95% CI: 12.5–18.9) months, respectively. T-DXd showed a numerically longer mPFS compared to chemotherapy (15.7 vs. 7.0 months, exploratory *P* = 0.020), chemotherapy plus pan-HER TKIs (15.7 vs. 7.0 months, exploratory *P* < 0.001), and pan-HER TKIs alone (15.7 vs. 4.2 months, exploratory *P* < 0.001).

In the *HER2*-*EGFR* co-altered subgroup (*n* = 19), treatments were as follows: chemotherapy in 2 cases, chemotherapy plus EGFR-TKIs in 3 cases, chemotherapy combined with dacomitinib in 2 cases, and EGFR-TKI monotherapy in 12 cases. The ORR in patients receiving chemotherapy plus EGFR-TKI therapy versus those receiving EGFR-TKI monotherapy were 100% and 33.3%, respectively. The DCRs were 100% and 91.7%. And the mPFS were 13.6 (95% CI, 9.1–18.0) months and 9.7 (95% CI, 5.9–13.5) months (exploratory *P* = 0.182). These results were largely consistent with the original analysis.

#### *HER2*-*EGFR* co-alteration detected by tissue NGS

To evaluate the impact of plasma definition on the results, we conducted a sensitivity analysis, retaining only cases with tissue-confirmed co-alterations (*n* = 17) (Supplement Table S3). In this analysis, the mPFS was 13.6 (95% CI: 9.1–18.0) months in the EGFR-TKI combined with chemotherapy group (*n* = 3) and 9.7 (95% CI: 6.7–12.7) months in the EGFR-TKI monotherapy group (*n* = 8) (exploratory *P* = 0.985). After excluding the 7 cases defined based on plasma criteria, the primary conclusions remained substantially unchanged.

### Safety and TRAEs

We categorised treatment regimens into a Chemotherapy group (chemotherapy alone, C + I, C + A, and C + I+A) and a Targeted therapy group (TKIs, C + T, and ADC) and analysed each group separately. AEs in patients receiving chemotherapy and targeted therapy were summarised in detail and are presented in Table 3. Overall, all-grade TRAEs occurring in ≥ 5% of patients included hepatic impairment, nausea, diarrhoea, leukopenia, and rash. Grade 3–4 AEs occurred in 11 patients (15.9%), including hepatic impairment, diarrhoea, leukopenia, neutropenia, thrombocytopenia, anaemia, lower-limb oedema, hypertension, proteinuria, and checkpoint inhibitor pneumonitis (CIP). Immune-related AEs occurred in 2 patients (2.9%), both in the chemotherapy group.

**Table 3:** Summary of TEAEs for patients harboring *HER2* alterations

		Chemotherapy (*n* = 29)			Targeted Therapy (*n* = 40)					
					Targeted Therapy (*n* = 40)			ADCs (*n* = 8)		
Parameters	Overall (*N* = 69)	Any grade	Grade 3–4	Death	Any grade	Grade 3–4	Death	Any grade	Grade 3–4	Death
Digestive system	25(36.2)	14(48.3)	0	0	11(27.5)	2(5.0)	0	3(37.5)	0	0
Hepatic Impairment	9(13.0)	6	0	0	3	1(2.5)	0	2	0	0
Nausea	8(11.6)	5	0	0	3	0	0	1	0	0
Diarrhea	4(5.8)	1	0	0	3	1(2.5)	0	0	0	0
Asthenia	3(4.3)	2	0	0	1	0	0	0	0	0
Decreased appetite	1(1.4)	0	0	0	1	0	0	0	0	0
Blood system	18(26.1)	10(34.5)	4(13.8)	0	8(20.0)	1(2.5)	0	3(37.5)	0	0
Leukopenia	11(15.9)	6	1(3.4)	0	5	0	0	1	0	0
Neutropenia	1(1.4)	1	1(3.4)	0	0	0	0	0	0	0
Monocytopenia	1(1.4)	0	0	0	1	0	0	1	0	0
Lymphopenia	1(1.4)	0	0	0	1	0	0	1	0	0
Thrombocytopenia	2(2.9)	1	1(3.4)	0	1	1(2.5)	0	0	0	0
Anemia	2(2.9)	2	1(3.4)	0	0	0	0	0	0	0
Skin, Mucosa, and Hair	10(14.5)	3(10.3)	0	0	7(17.5)	1(2.5)	0	1(12.5)	0	0
Mucositis oral	2(2.9)	0	0	0	2	0	0	0	0	0
Rash	4(5.8)	1	0	0	3	0	0	0	0	0
Alopecia	3(4.3)	2	0	0	1	0	0	1	0	0
Lower Limb Edema	1(1.4)	0	0	0	1	1(2.5)	0	0	0	0
Circulatory System	4(5.8)	0	0	0	3(7.5)	1(2.5)	0	1(12.5)	0	0
Hypertension	2(2.9)	0	0	0	2	1(2.5)	0	0	0	0
Myocarditis	2(2.9)	0	0	0	1	0	0	1	0	0
Urinary System	2(2.9)	0	0	0	2(5.0)	1(2.5)	0	0	0	0
Hematuria	1(1.4)	0	0	0	1	0	0	0	0	0
Proteinuria	1(1.4)	0	0	0	1	1(2.5)	0	0	0	0
Additional TRAEs of special interest	2(2.9)	2(6.9)	1(3.4)	0	0	0	0	0	0	0
Checkpoint Inhibitor Pneumonitis (CIP)	1(1.4)	1	1(3.4)	0	0	0	0	0	0	0
Checkpoint Inhibitor Colitis (CIC)	1(1.4)	1	0	0	0	0	0	0	0	0

In the chemotherapy group (*n* = 29), gastrointestinal AEs were most common (*n* = 14), followed by haematological AEs (*n* = 10). Grade 3–4 AEs occurred in 5 patients (17.2%), primarily haematological, with only one case of CIP. No treatment-related deaths occurred. In the targeted therapy group (*n* = 40), AEs were distributed across gastrointestinal (*n* = 11), haematological (*n* = 8), mucocutaneous (*n* = 7), and cardiovascular/urinary systems (*n* = 5). Grade 3–4 events occurred in 6 patients (15.0%) and were distributed across the gastrointestinal, haematological, mucocutaneous, cardiovascular, and urinary systems. No immune-related AEs or treatment-related deaths were observed. Furthermore, among the 8 ADC-treated patients, all AEs were grade 1–2, predominantly haematological (*n* = 5; 62.5%).

Among the 69 patients, two discontinued therapy due to TRAEs; both received the C + I regimen and were discontinued for grade 3 hematologic AEs: one case of thrombocytopenia and one of leukopenia. No treatment-related discontinuation events occurred in the targeted therapy group (*n* = 40), particularly in the ADC group (*n* = 8).

In summary, most events were preventable or manageable with appropriate monitoring and intervention.

### An illustrative case of durable response to first-line ADC

A 55-year-old female with stage IV lung adenocarcinoma harboring a *HER2* exon 20 insertion mutation (without *EGFR* co-mutation) and a PD-L1 TPS of < 1% received first-line therapy with T-DXd (Supplement Figure S2). At baseline, her ECOG performance status was 1, and she reported symptoms including fatigue, chest tightness, and cough. Baseline imaging revealed lung cancer of the right middle lobe. After 2 cycles, the first on-treatment scan demonstrated a PR; her tumor‑related symptoms (fatigue, chest tightness, and cough) notably improved, and her ECOG status remained at 1. The response deepened at the 4-cycle assessment. The patient maintained this response for over 12 months before radiological progression occurred. Treatment was well‑tolerated with only Grade 2 leukopenia.

## Discussion

This study delineated the clinicopathologic features and outcomes of *HER2*-altered advanced NSCLC. We confirmed that the clinical profile of this population included younger age, female predominance, adenocarcinoma histology, non-smoking history, and moderate-poor differentiation, aligning with prior epidemiology [26]. NGS analysis showed that *HER2* alterations were predominantly exon 20 insertions and largely mutually exclusive with other drivers. *TP53* and *EGFR* were the most frequent co-mutations.

Prior first-line real-world data in *HER2*-altered NSCLC were limited to chemotherapy or pan-HER TKIs. This study was the first to include T-DXd in this comparison. For patients with only *HER2* alterations, chemotherapy-based regimens yielded an mPFS of 7.6 months, confirming an unmet need. First-line T-DXd therapy was associated with a numerically longer mPFS of 15.7 months compared with chemotherapy (7.6 months), TKIs (4.4 months), or C + T (7.0 months). The numerically lower mPFS observed with Pan-*HER* TKI monotherapy (4.4 months) compared with chemotherapy-based treatment (7.6 months) should be interpreted cautiously, as confounding factors may fully explain the difference. However, the outcome data might differ with newer-generation *HER2* TKIs such as Zongertinib and Sevabertinib. Prospective trials are needed to confirm the efficacy and safety of first-line T-DXd. Subsequent research should explore T-DXd-based combinations with chemotherapy, ICIs, or antiangiogenic agents to overcome or delay resistance. Future studies are also needed to identify subgroups with exceptional responses or primary resistance to T-DXd therapy, ultimately enabling personalised, precision-based treatment.

The treatment landscape for *HER2*-mutant advanced NSCLC has evolved rapidly during the preparation of this manuscript. Zongertinib (BI1810631), a highly selective oral *HER2* tyrosine kinase inhibitor, received FDA accelerated approval for previously treated *HER2*-mutant NSCLC in August 2025 [15] and subsequently gained first-line approval of NMPA for *HER2* TKD-mutant advanced NSCLC in May 2026. The NCCN guidelines have incorporated zongertinib as a preferred subsequent therapy option and, as of March 2026, as a first-line treatment option [27]. In the phase Ib Beamion LUNG-1 trial, first-line zongertinib demonstrated an ORR of 76% in treatment-naïve *patients with advanced HER2-mutant NSCLC*,* with an* mPFS of 14.4 months and a manageable safety profile [14]. The ongoing phase III Beamion LUNG-2 trial (NCT06151574) is directly comparing zongertinib versus standard-of-care chemotherapy in the first-line setting. Our real-world data showed the efficacy of first-line T-DXd in *HER2*-altered NSCLC, with an mPFS of 15.7 months in the only *HER2*-altered subgroup. However, with the emergence of oral *HER2* TKIs offering convenient once-daily dosing and favourable safety profiles, the role of T-DXd in the first-line setting remains uncertain, and the optimal sequencing and positioning of T-DXd versus HER2-TKIs in the first-line treatment algorithm remain open questions requiring future head-to-head comparative studies. Importantly, the pan-HER TKIs used in our cohort (afatinib, dacomitinib) were distinct from the newer selective HER2 TKIs. Thus, our observed poor outcomes with pan-HER TKI monotherapy should not be extrapolated to agents such as zongertinib or sevabertinib.

Previous studies often excluded patients with *HER2*-*EGFR* co-alterations during screening or did not specify their status. Thus, the optimal treatment strategy for this specific subgroup remained unexplored. In our cohort, first-line therapy predominantly involved TKIs, especially EGFR-TKIs. However, our results showed that the mPFS with third-generation EGFR-TKIs monotherapy was only 10.2 months, which was notably shorter than the mPFS reported for *EGFR*-mutant patients [22]. This suggested that pre-existing activation of the dual HER2/EGFR pathway might accelerate resistance to third-generation EGFR-TKIs. Although first-generation EGFR-TKI outcomes appeared comparable to historical controls, small sample sizes limit conclusions. Prior research [28] highlighted the critical role of *EGFR*/*HER2* heterodimers in oncogenic signalling. *HER2*, with its constitutively “open” active conformation, serves as a preferred dimerisation partner, significantly enhancing the efficiency and stability of ligand-dependent and -independent signalling. We therefore hypothesised that this primary dual-pathway activation might establish a signalling network with higher activity and greater compensatory potential. Targeting *EGFR* alone may allow rapid escape via the HER2 pathway, while broad inhibition with pan-HER TKIs might be insufficient to effectively suppress both pathways, a possibility that requires further investigation. The FLAURA2 trial supported combination strategies in *EGFR*-mutant disease. Chemo plus EGFR-TKI showed a longer PFS in patients with *HER2*-*EGFR* co-alterations. However, given that only 3 cases were included in the combined chemotherapy group, the statistical power was severely insufficient. Consequently, larger prospective studies are needed to define the optimal first-line approach for this population. Notably, our study included no cases of first-line T-DXd treatment for *HER2*-*EGFR* co-altered patients. Exploring the efficacy of *HER2*-targeted ADCs (alone or combined with EGFR-TKIs) or novel bispecific ADCs in this subgroup represents a highly promising direction for future research.

Our subgroup analysis showed that our conclusions remain robust in both populations with pure *HER2* mutations and those with *HER2* exon 20 insertion mutations. In the population with only *HER2* alterations, we did not observe an impact of PD-L1 expression levels on prognosis. However, the presence of *TP53* mutations was associated with poor prognosis in this group. Prior evidence [29] suggested that co-occurring *EGFR* and *TP53* mutations may promote genome doubling and targeted therapy resistance. Our findings indicated that, in HER2-altered NSCLC, this TP53-associated resistance mechanism may also operate.

We analysed TRAEs in patients receiving chemotherapy and targeted therapies. The results showed that TRAEs in the chemotherapy group primarily affected the digestive system, whereas severe (≥ Grade 3) TRAEs were predominantly haematological (4/6). Furthermore, both instances of immune-related AEs occurred in this group. The targeted therapy group also experienced mainly gastrointestinal TRAEs, but with a broader systemic distribution, including skin/mucosa, cardiovascular, and urinary involvement. Severe TRAEs in this group were primarily gastrointestinal. A separate analysis of TRAEs in patients receiving first-line ADC therapy revealed that all events were < Grade 3, with the majority being haematological AEs (5/10). These findings must be interpreted with extreme caution, as they are likely a statistical artefact of the extremely small sample size and do not accurately represent the established toxicity profile of T-DXd, which includes significant rates of myelosuppression, nausea, and a notable risk of potentially fatal ILD, as documented in the large, prospective DESTINY-Lung01 and DESTINY-Lung02 trials.

Our study had several limitations. First, the retrospective design, combined with the molecular heterogeneity of *HER2* alterations and small sample sizes, introduced inherent reporting bias and limited the reliability of conclusions. Second, efficacy assessments were based on retrospective review of clinical radiology reports without independent central review, which may introduce variability in response and progression dating. Third, the inclusion of both stage IIIB/IIIC and IV patients, while sensitivity analyses confirm consistent findings after excluding stage III cases, might still limit generalizability to the purely metastatic population. Fourth, mOS could not be calculated for individual treatment groups due to insufficient deaths (*n* = 27) at data cutoff, and only 1‑year and 2‑year survival rates were reported.

Selection bias was a major concern. Patients with more aggressive disease might have been preferentially treated with chemotherapy, potentially underestimating its efficacy relative to T‑DXd, while T‑DXd became available only after its 2024 approval, introducing potential lead‑time bias. Although baseline characteristics by treatment group are provided, unmeasured confounding could not be excluded. For plasma‑defined *HER2*‑*EGFR* co‑alterations (*n* = 7), VAF data were unavailable for clonality assessment. These cases were excluded from sensitivity analyses, and co‑alteration subgroup findings were primarily based on tissue‑confirmed cases. PD‑L1 testing was incomplete and non-standardised. Despite exploratory analyses showing no significant association with PFS, the small sample size and missing data limit the reliability of these findings, warranting caution when interpreting immunotherapy‑containing regimens. Additionally, the fixed timing of initial imaging assessments precluded precise determination of time to first response.

Finally, this study was conducted before the first‑line approval of zongertinib. Therefore, our TKI efficacy data, based on pan-HER TKIs (afatinib, dacomitinib), should not be extrapolated to newer selective HER2 TKIs, which now offer a convenient oral first‑line option for *HER2* TKD‑mutant NSCLC. Despite these limitations, this study provided valuable real‑world evidence on first‑line treatment outcomes in *HER2*‑altered NSCLC, particularly in the Chinese population.

## Conclusions

In summary, this study indicated that first-line T-DXd therapy showed promising activity signals in patients with only *HER2*-altered NSCLC. Additionally, the treatment needs of patients with *HER2*-*EGFR* co-alterations remained far from adequately addressed. Future research, particularly prospective studies, is warranted to validate these findings in the broader population of patients with *HER2*-altered NSCLC.

## Supplementary Information

Supplementary Material 1: Supplement Table S1: Baseline characteristics of HER2-altered NSCLC patients.

Supplementary Material 2: Supplement Table S2: Details in only the HER2 alteration group.

Supplementary Material 3: Supplement Table S3: Detailed characteristics of HER2-EGFR co-alteration cases.

Supplementary Material 4: Supplement Table S4: Baseline by treatment group in only the HER2-altered group.

Supplementary Material 5: Supplement Figure S1. Kaplan-Meier estimates of PFS in the first-line setting. (A). PFS in patients harboring only HER2 mutations receiving chemotherapy, ADC, TKIs, and chemotherapy combined with TKIs (C+T). (B). PFS in patients harboring only HER2 exon 20 insertion mutation receiving chemotherapy, ADC, TKIs, and chemotherapy combined with TKIs (C+T). (C). PFS in patients harboring HER2-EGFR co-alterations with or without concurrent HER2 amplification. (D). PFS in patients harboring only HER2 alteration with or without PD-L1 expression. (E). PFS in patients harboring only HER2 alteration with or without TP53 mutation. (F). PFS in patients harboring HER2-EGFR co-alteration with or without TP53 mutation.

Supplementary Material 6: Supplement Figure S2. An illustrative case of durable response to first-line T-DXd.

## Data Availability

Data are available upon reasonable request. The data supporting this study’s findings are available from the corresponding author upon reasonable request.
