# Supplementary material for: First-line treatment outcomes in HER2-altered advanced NSCLC: a real-world multi-center study comparing trastuzumab deruxtecan with conventional therapies and analysis by HER2/EGFR co-alteration status
Source: BMC Cancer. 2026 Oct 12;26:1220. doi: 10.1186/s12885-026-16688-7 (PMC13660007; doi:10.1186/s12885-026-16688-7)
Supplement: Supplementary file 1 [file 12885_2026_16688_MOESM1_ESM.docx]

**Supplement Table S1: Stage-stratified survival outcomes and sensitivity analysis excluding Stage III patients.**

|  | Only HER2 alterations (n=37) | | | | HER2-EGFR co-alterations (n=19) | |
| --- | --- | --- | --- | --- | --- | --- |
|  | Chemotherapy-based therapy (n=20) | Chemotherapy + pan-HER TKIs (n=4) | Pan-HER TKIs (n=5) | T-DXd (n=8) | Chemotherapy + EGFR-TKIs (n=3) | EGFR-TKIs (n=12) |
| Best Response, n (%) |  |  |  |  |  |  |
| CR | 0 | 0 | 0 | 0 | 0 | 0 |
| PR | 7 | 0 | 0 | 7 | 3 | 4 |
| SD | 10 | 4 | 4 | 1 | 0 | 7 |
| PD | 3 | 0 | 1 | 0 | 0 | 1 |
| ORR, % | 35.0 | 0.0 | 0.0 | 87.5 | 100.0 | 33.3 |
| DCR, % | 85.0 | 100.0 | 80.0 | 100.0 | 100.0 | 91.7 |
| PFS |  |  |  |  |  |  |
| Events, n (%) | 16 (80.0) | 4 (100.0) | 4 (80.0) | 4 (50.0) | 3 (100.0) | 11 (91.7) |
| Median, months (95% CI) | 7.6 (6.6-8.5) | 7.0 (2.9-11.1) | 4.2 (3.4–5.1) | 15.7 (12.5-18.9) | 13.6 (9.1-18.0) | 9.7 (5.9-13.5) |
| One-year survival rate, % | 19 (95.0) | 3 (75.0) | 5 (100.0) | 8 (100.0) | 3 (100.0) | 12 (100.0) |
| Two-year survival rate, % | 18 (90.0) | 3 (75.0) | 1(20.0) | NE | 3 (100.0) | 11 (91.7) |
