# Supplementary material for: First-line treatment outcomes in HER2-altered advanced NSCLC: a real-world multi-center study comparing trastuzumab deruxtecan with conventional therapies and analysis by HER2/EGFR co-alteration status
Source: BMC Cancer. 2026 Oct 12;26:1220. doi: 10.1186/s12885-026-16688-7 (PMC13660007; doi:10.1186/s12885-026-16688-7)
Supplement: Supplementary file 2 [file 12885_2026_16688_MOESM2_ESM.docx]

**Supplement Table S2: Details in only *HER2* alteration group.**

| ID | *HER2* alteration subtype | Sample type | Treatment | PFS (months) | Status | Best of response |
| --- | --- | --- | --- | --- | --- | --- |
| 1 | Exon 20 insertion mutation | Tissue | Chemotherapy-based | 6.9 | 1 | SD |
| 2 | Exon 20 insertion mutation | Tissue | Chemotherapy-based | 7.6 | 1 | SD |
| 3 | Exon 20 insertion mutation | Tissue | Chemotherapy-based | 7.8 | 1 | PR |
| 4 | Missense mutations | Plasma | Chemotherapy-based | 44.0 | 1 | PR |
| 5 | Exon 20 insertion mutation | Tissue | Chemotherapy-based | 8.5 | 1 | SD |
| 6 | Exon 20 insertion mutation | Plasma | Pan-HER TKI | 5.9 | 1 | SD |
| 7 | Exon 20 insertion mutation + amplification | Plasma | Pan-HER TKI | 4.4 | 1 | SD |
| 8 | Exon 20 insertion mutation | Plasma | Pan-HER TKI | 3.1 | 0 | SD |
| 9 | Missense mutations | Tissue | Chemotherapy-based | 5.1 | 1 | SD |
| 10 | Exon 20 insertion mutation | Tissue | Chemotherapy-based | 7.1 | 1 | SD |
| 11 | Exon 20 insertion mutation + amplification | Tissue | Chemotherapy-based | 3.4 | 1 | PR |
| 12 | Exon 20 insertion mutation | Tissue | Chemotherapy-based | 18.0 | 1 | SD |
| 13 | Exon 20 insertion mutation | Tissue | Chemotherapy-based | 9.8 | 0 | SD |
| 14 | Exon 20 insertion mutation | Tissue | Chemotherapy-based | 7.5 | 0 | PR |
| 15 | Exon 20 insertion mutation | Tissue | Chemo + pan-HER TKI | 7.0 | 1 | SD |
| 16 | Exon 20 insertion mutation | Tissue | Chemotherapy-based | 1.5 | 1 | PD |
| 17 | Missense mutations | Plasma | Chemotherapy-based | 3.3 | 0 | SD |
| 18 | Exon 20 insertion mutation | Tissue | Pan-HER TKI | 5.3 | 1 | SD |
| 19 | Exon 20 insertion mutation | Tissue | Chemotherapy-based | 2.1 | 0 | SD |
| 20 | Exon 20 insertion mutation | Tissue | Chemotherapy-based | 1.9 | 1 | PD |
| 21 | Splice-site mutation | Tissue | Chemotherapy-based | 5.6 | 1 | PR |
| 22 | Exon 20 insertion mutation | Tissue | Chemo + pan-HER TKI | 3.4 | 1 | SD |
| 23 | Exon 20 insertion mutation | Tissue | T-DXd | 11.9 | 1 | PR |
| 24 | Missense mutations | Plasma | Chemotherapy-based | 15.2 | 1 | PR |
| 25 | Unknown mutation subtype | Tissue | Pan-HER TKI | 4.2 | 1 | SD |
| 26 | Exon 20 insertion mutation + amplification | Tissue | Pan-HER TKI | 3.6 | 1 | PD |
| 27 | Exon 20 insertion mutation | Tissue | Chemotherapy-based | 7.1 | 1 | SD |
| 28 | Exon 20 insertion mutation | Tissue | T-DXd | 16.8 | 1 | PR |
| 29 | Fusion mutation | Tissue | T-DXd | 13.6 | 1 | PR |
| 30 | Missense mutations + amplification | Tissue | Chemotherapy-based | 7.4 | 1 | SD |
| 31 | Exon 20 insertion mutation | Tissue | T-DXd | 14.6 | 0 | PR |
| 32 | Exon 20 insertion mutation | Plasma | T-DXd | 8.7 | 0 | SD |
| 33 | Exon 20 insertion mutation | Plasma | T-DXd | 5.3 | 0 | PR |
| 34 | Exon 20 insertion mutation | Plasma | T-DXd | 12.3 | 0 | PR |
| 35 | Exon 20 insertion mutation | Tissue | Chemotherapy-based | 3.9 | 0 | SD |
| 36 | Unknown mutation subtype | Plasma | Chemotherapy-based | 14.7 | 1 | PR |
| 37 | Exon 20 insertion mutation | Tissue | Chemotherapy-based | 3.9 | 1 | SD |
| 38 | Splice-site mutation + amplification | Tissue | Chemotherapy-based | 10.9 | 1 | SD |
| 39 | Exon 20 insertion mutation | Tissue | Chemo + pan-HER TKI | 7.6 | 1 | SD |
| 40 | Missense mutations | Tissue | Chemotherapy-based | 8.7 | 1 | SD |
| 41 | Pure amplification | Tissue | Chemotherapy-based | 1.8 | 1 | PD |
| 42 | Pure amplification | Tissue | Chemotherapy-based | 8.7 | 1 | PR |
| 43 | Pure amplification | Tissue | Chemo + pan-HER TKI | 8.7 | 1 | SD |
| 44 | Pure amplification | Tissue | Chemotherapy-based | 7.2 | 1 | PR |
| 45 | Pure amplification | Tissue | T-DXd | 15.7 | 1 | PR |
