# Supplementary material for: First-line treatment outcomes in HER2-altered advanced NSCLC: a real-world multi-center study comparing trastuzumab deruxtecan with conventional therapies and analysis by HER2/EGFR co-alteration status
Source: BMC Cancer. 2026 Oct 12;26:1220. doi: 10.1186/s12885-026-16688-7 (PMC13660007; doi:10.1186/s12885-026-16688-7)
Supplement: Supplementary file 3 [file 12885_2026_16688_MOESM3_ESM.docx]

**Supplement Table S3: Detailed characteristics of *HER2-EGFR* co-alteration cases.**

| ID | Sample type | *EGFR* mutation subtype | *HER2* alteration subtype | Same sample | Treatment | PFS (months) | Status | Best of response |
| --- | --- | --- | --- | --- | --- | --- | --- | --- |
| 1 | Plasma | Exon 21 L858R mutation | Missense mutation | Yes | EGFR-TKI | 17.3 | 1 | PR |
| 2 | Tissue | Exon 21 L858R mutation | Missense mutation | Yes | EGFR-TKI | 11.4 | 1 | PR |
| 3 | Plasma | Exon 19 deletion mutation | Undetermined mutation subtype | Yes | EGFR-TKI | 10.2 | 1 | SD |
| 4 | Tissue | Exon 21 L858R mutation | Fusion mutation | Yes | Chemo + EGFR-TKI | 21.5 | 1 | PR |
| 5 | Plasma | Other mutation subtypes | Exon 20 insertion mutation | Yes | EGFR-TKI | 1.6 | 0 | SD |
| 6 | Tissue | Other mutation subtypes | Undetermined mutation subtype | Yes | Chemotherapy | 4.6 | 1 | SD |
| 7 | Tissue | Exon 21 L858R mutation | Splice-site mutation | Yes | Chemo + EGFR-TKI | 13.6 | 1 | PR |
| 8 | Plasma | Exon 21 L858R mutation | Missense mutation | Yes | EGFR-TKI | 9.7 | 1 | PR |
| 9 | Tissue | Exon 21 L858R mutation | Missense mutation | Yes | EGFR-TKI | 9.7 | 1 | PR |
| 10 | Plasma | Exon 21 L858R mutation | Undetermined mutation subtype | Yes | EGFR-TKI | 14.5 | 1 | SD |
| 11 | Tissue | Exon 19 deletion mutation | Missense mutation | Yes | EGFR-TKI | 6.6 | 1 | SD |
| 12 | Tissue | Exon 21 L858R mutation | Undetermined mutation subtype | Yes | Chemotherapy | 27.4 | 1 | PR |
| 13 | Tissue | Exon 21 L858R mutation | Undetermined mutation subtype | Yes | Chemo + pan-HER TKI | 13.6 | 1 | PR |
| 14 | Tissue | Exon 21 L858R mutation | Missense mutation | Yes | Pan-HER TKI | 32.4 | 1 | SD |
| 15 | Tissue | Exon 19 deletion mutation | Missense mutation | Yes | EGFR-TKI | 14.4 | 1 | PR |
| 16 | Plasma | Exon 21 L858R mutation | Missense mutation | Yes | EGFR-TKI | 2.9 | 1 | SD |
| 17 | Tissue | Exon 21 L858R mutation | Missense mutation | Yes | Pan-HER TKI | 2.5 | 1 | PD |
| 18 | Tissue | Other mutation subtypes | Undetermined mutation subtype | Yes | Chemo + pan-HER TKI | 6.8 | 1 | SD |
| 19 | Tissue | Exon 21 L858R mutation | Pure amplification | Yes | EGFR-TKI | 6.7 | 1 | PD |
| 20 | Tissue | Exon 21 L858R mutation | Pure amplification | Yes | EGFR-TKI | 9.3 | 1 | SD |
| 21 | Plasma | Exon 21 L858R mutation | Pure amplification | Yes | EGFR-TKI | 3.2 | 1 | SD |
| 22 | Tissue | Exon 21 L858R mutation | Pure amplification | Yes | Chemo + EGFR-TKI | 10.8 | 1 | PR |
| 23 | Tissue | Exon 21 L858R mutation | Pure amplification | Yes | EGFR-TKI | 33.5 | 1 | SD |
| 24 | Tissue | Exon 21 L858R mutation | Pure amplification | Yes | EGFR-TKI | 28.5 | 1 | PR |
