# Supplementary material for: First-line treatment outcomes in HER2-altered advanced NSCLC: a real-world multi-center study comparing trastuzumab deruxtecan with conventional therapies and analysis by HER2/EGFR co-alteration status
Source: BMC Cancer. 2026 Oct 12;26:1220. doi: 10.1186/s12885-026-16688-7 (PMC13660007; doi:10.1186/s12885-026-16688-7)
Supplement: Supplementary file 4 [file 12885_2026_16688_MOESM4_ESM.docx]

**Supplement Table S4: Baseline by treatment group in only *HER2*-altered group.**

| Characteristics | C+I (n=11) | C+A (n=8) | C+I+A (n=7) | Chemo+pan-HER TKI (n=4) | Pan-HER TKI (n=6) | T-DXd (n=8) | P value |
| --- | --- | --- | --- | --- | --- | --- | --- |
| Sex |  |  |  |  |  |  |  |
| Male | 4(36.4) | 4(50.0) | 3(42.9) | 1(25.0) | 2(33.3) | 3(37.5) | 0.983 |
| Female | 7(63.6) | 4(50.0) | 4(57.1) | 3(75.0) | 4(66.7) | 5(62.5) |  |
| Year |  |  |  |  |  |  | 0.774 |
| Y≤50 | 4(36.4) | 0(0) | 2(28.6) | 1(25.0) | 2(33.3) | 1(12.5) |  |
| 50<Y≤65 | 6(54.5) | 5(62.5) | 4(57.1) | 2(50.0) | 3(50.0) | 4(50.0) |  |
| Y>65 | 1(9.1) | 3(37.5) | 1(14.3) | 1(25.0) | 1(16.7) | 3(37.5) |  |
| Smoking history |  |  |  |  |  |  | 0.235 |
| Never | 7(63.6) | 4(50.0) | 7(100.0) | 4(100.0) | 5(83.3) | 6(75.0) |  |
| Current/former | 4(36.4) | 4(50.0) | 0(0) | 0(0) | 1(16.7) | 2(25.0) |  |
| ECOG |  |  |  |  |  |  | 0.201 |
| 0 | 1(9.1) | 2(25.0) | 2(28.6) | 2(50.0) | 0(0) | 2(25.0) |  |
| 1 | 5(45.5) | 6(75.0) | 4(57.1) | 2(50.0) | 4(66.7) | 6(75.0) |  |
| 2 | 5(45.5) | 0(0) | 1(14.3) | 0(0) | 1(16.7) | 0(0) |  |
| 3 | 0(0) | 0(0) | 0(0) | 0(0) | 1(16.7) | 0(0) |  |
| Pathology |  |  |  |  |  |  | 0.75 |
| Adenocarcinoma | 11(100.0) | 8(100.0) | 7(100.0) | 4(100.0) | 6(100.0) | 7(87.5) |  |
| Large cell carcinoma | 0(0) | 0(0) | 0(0) | 0(0) | 0(0) | 1(12.5) |  |
| Differentiation |  |  |  |  |  |  | 0.476 |
| NA | 4(36.4) | 3(37.5) | 5(71.4) | 3(75.0) | 4(66.7) | 3(37.5) |  |
| poorly differentiation | 2(18.2) | 3(37.5) | 0(0) | 0(0) | 2(33.3) | 2(25.0) |  |
| Moderately to poorly differentiated | 3(27.3) | 1(12.5) | 2(28.6) | 0(0) | 0(0) | 0(0) |  |
| Moderately differentiation | 1(9.1) | 1(12.5) | 0(0) | 1(25.0) | 0(0) | 3(37.5) |  |
| Moderately to well differentiated | 1(9.1) | 0(0) | 0(0) | 0(0) | 0(0) | 0(0) |  |
| Metastases |  |  |  |  |  |  |  |
| Lung | 9(81.8) | 7(87.5) | 5(71.4) | 4(100.0) | 5(83.3) | 7(87.5) | 0.958 |
| Bone | 5(45.5) | 2(25.0) | 1(14.3) | 1(25.0) | 2(33.3) | 6(75.0) | 0.22 |
| Liver | 2(18.2) | 1(12.5) | 1(14.3) | 1(25.0) | 2(33.3) | 3(37.5) | 0.822 |
| Brain | 2(18.2) | 1(12.5) | 1(14.3) | 1(25.0) | 0(0) | 3(37.5) | 0.662 |
| Pleural effusion | 1(9.1) | 2(25.0) | 2(28.6) | 2(50.0) | 2(33.3) | 2(25.0) | 0.657 |
| UICC stage |  |  |  |  |  |  | 0.349 |
| ⅢB/ⅢC | 1(9.1) | 2(25.0) | 4(57.1) | 0(0) | 1(16.7) | 0(0) |  |
| ⅣA | 3(27.3) | 3(37.5) | 2(28.6) | 2(50.0) | 3(50.0) | 3(37.5) |  |
| ⅣB | 7(63.6) | 3(37.5) | 1(14.3) | 2(50.0) | 2(33.3) | 5(62.5) |  |
| HER2 mutations |  |  |  |  |  |  | 0.03 |
| NA | 0(0) | 1(12.5) | 1(14.3) | 0(0) | 1(16.7) | 0(0) |  |
| EX20ins mutations | 9(81.8) | 1(12.5) | 4(57.1) | 3(75.0) | 5(83.3) | 6(75.0) |  |
| Missense mutations | 2(18.2) | 2(25.0) | 2(28.6) | 0(0) | 0(0) | 0(0) |  |
| Fusion mutations | 0(0) | 0(0) | 0(0) | 0(0) | 0(0) | 1(12.5) |  |
| Splice-site mutations | 0(0) | 1(12.5) | 0(0) | 0(0) | 0(0) | 1(12.5) |  |
| *HER2* amplification | 0(0) | 4(50.0) | 1(14.3) | 1(25.0) | 2(33.3) | 1(12.5) | 0.092 |
| PD-L1 expression |  |  |  |  |  |  | 0.332 |
| NA | 5(45.5) | 4(50.0) | 2(28.6) | 1(25.0) | 2(33.3) | 4(50.0) |  |
| Negative | 4(36.4) | 2(25.0) | 2(28.6) | 3(75.0) | 0(0) | 2(25.0) |  |
| 1%≤ <50% | 0(0) | 2(25.0) | 3(42.9) | 0(0) | 3(50.0) | 2(25.0) |  |
| ≥50% | 2(18.2) | 0(0) | 0(0) | 0(0) | 1(16.7) | 0(0) |  |
| *HER2* alteration partner |  |  |  |  |  |  |  |
| *TP53* | 3(27.3) | 4(50.0) | 4(57.1) | 3(75.0) | 1(16.7) | 2(25.0) | 0.391 |
| Cell cycle-related gene mutations | 2(18.2) | 4(50.0) | 1(14.3) | 1(25.0) | 0(0) | 0(0) | 0.123 |
